# Explainable Artificial Intelligence for Coffee Quality Control: From Coffee Origins to Aroma Intensity

**DOI:** 10.3390/foods15091543

**Published:** 2026-04-29

**Authors:** Giorgio Felizzato, Eloisa Bagnulo, Giorgia Botta, Giulia Tapparo, Chiara Cordero, Luciano Navarini, Cecilia Cagliero, Erica Liberto, Andrea Caratti

**Affiliations:** 1 Dipartimento di Scienza e Tecnologia del Farmaco, Università di Torino, Via Pietro Giuria 9, 10125 Torino, Italy; 2 Illycaffè S.p.A., Via Flavia 110, 34147 Trieste, Italy

**Keywords:** coffee quality, specialty coffee, origin identity, SHAP, aroma intensity, explainable AI

## Abstract

Background: Coffee quality is strongly influenced by origin-related factors, or terroir, which shape chemical composition and sensory characteristics. In the specialty coffee sector, where authenticity, traceability, and flavour distinctiveness drive value, understanding the molecular basis of sensory attributes, particularly perceived intensity, is essential. Methods: This study combined analytical chemistry and explainable artificial intelligence to explore relationships between volatile composition, coffee origin, and sensory intensity. Roasted and ground single-origin coffees from five provenances were analysed using headspace solid-phase microextraction coupled with gas chromatography–mass spectrometry (HS-SPME/GC–MS). A Support Vector Machine (SVM) classifier discriminated coffee origins based on volatile profile, and SHapley Additive exPlanations (SHAP) identified key compounds. Ridge Regression (RR) was applied to predict sensory intensity values assigned by an expert panel. Results: The SVM model classified coffee origins with 91% accuracy, and SHAP analysis highlighted the volatiles most responsible for differentiation. RR predicted sensory intensity with R^2^ = 0.88 and RMSE = 0.38, linking molecular profiles with panel-assigned intensity scores. Conclusions: This approach connects molecular profile with packaging-declared aroma intensity, offering an indirect yet informative link to sensory perception and illustrating the potential of data-driven methods in sensory science. Overall, the proposed explainable AI approach provides a transparent and reproducible connection between chemical composition, sensory traits, and perceived quality. This strategy supports more objective and traceable quality assessment systems, aligning analytical precision with sensory expertise, which is an essential step toward the evolution of quality control in industrial applications.

## 1. Introduction

Coffee is the second most traded commodity in the world, with an estimated 500 billion cups consumed every day [1]. The two main commercially relevant species are Arabica (*Coffea arabica* L.) and Robusta (*Coffea canephora* Pierre), with Arabica accounting for over 70% of global consumption due to its superior sensory quality. However, Arabica is more sensitive to climatic variations than Robusta, making it more vulnerable to the impacts of climate change. As a result, climate change is steadily reducing the areas suitable for its cultivation, lowering potential yields, and making the plants more prone to pests and diseases [2,3].

Generally, altitude, latitude, temperature, and rainfall strongly influence the chemical composition and sensory profile of coffee [4]. Moreover, the final quality of the beverage is shaped by the entire production chain, including geographical origin, species, harvesting practices, processing methods (especially roasting and grinding), storage, and brewing technique [5,6].

Growing consumer awareness of product quality and evolving tastes, together with the influence of global coffee culture, has driven the sustained growth of specialty coffee, making it a globally recognised trend and social phenomenon [7]. According to the Specialty Coffee Association of Europe, it is defined as “*a carefully crafted coffee-based beverage, recognised by consumers, within a limited market at a given time, for its unique quality, distinctive flavour, and character, setting it apart from standard coffee drinks*”. The beverage is made from beans grown in precisely defined regions and meets the highest standards in terms of green coffee quality, roasting, storage, and brewing [8]. Overall, coffee quality reflects the interaction between cultivation systems and environmental conditions. Consequently, the origin of single-origin coffees can be traced to specific regions, as their quality reflects the unique local environmental factors. The concept of *terroir*, therefore, links the beverage to its geographic and cultural context, while contributing to its value [9].

In addition to coffee terroir, coffee aroma intensity, defined by sensory panels based on strength, flavour, and aroma [10], is a key factor influencing consumer choice. Labelling aroma intensity shapes expectations, purchase intent, and satisfaction, as consumers show clear preferences for stronger or milder profiles [11,12]. Individual differences in taste sensitivity, including genetic variation in bitter and sweet taste receptors, influence preferences for coffee aroma intensity. More sensitive individuals tend to prefer sweeter, less bitter coffee, while others favour stronger, more bitter profiles [13,14].

Aroma intensity is closely linked to the degree of roasting. Roasting progressively transforms the chemical composition of coffee beans, altering the balance among acidity, bitterness, body, and aromatic compounds that together define the sensory perception of intensity in the final product [15].

Light roasts show higher acidity, floral–fruity notes, and higher chlorogenic acids and antioxidants, but lower body and bitterness. Medium roasts provide a balanced profile with high aromatic complexity and volatile release, often with caramel, nutty, and roasted notes. Dark roasts increase bitterness, body, and aftertaste while reducing acidity and sweetness. They also promote the formation of burnt or smoky flavours, which may become dominant or undesirable at extreme roasting levels [15,16,17].

Given the complexity of specialty coffee chemical and sensory profiles and their link to consumer preferences, advanced analytical and computational methods are increasingly needed to decode them [18,19]. Indeed, recently, machine learning has shown strong potential in identifying aroma patterns in complex food matrices [20,21]. For example, in cocoa, chromatographic fingerprints combined with supervised and unsupervised models enabled accurate origin classification, while in tea, AI-based sensomics allowed quantitative profiling of key sensory markers for quality control [22,23]. Overall, these studies demonstrate that combining analytical chemistry with machine learning can generate robust and reproducible flavour profiles for authentication and quality assessment.

However, the application of advanced machine learning and deep learning models is often limited by the lack of transparency, even though they usually achieve higher accuracy than less complex models. Behaving as “black boxes,” they make it difficult to clearly identify the chemical compounds or features driving the discrimination among different origins or quality states. This lack of interpretability poses a significant challenge when the goal is not only accurate classification but also understanding the molecular mechanisms underlying quality differences, which is essential for informed decision-making and corrective actions in industrial processes [24,25].

In an effort to mitigate the lack of interpretability of certain machine learning models and to augment human reasoning and decision-making, attention has been drawn to explainable artificial intelligence (XAI) approaches [26].

Within this context, SHapley Additive exPlanations (SHAP) has emerged as a powerful method; it quantifies the contribution of each feature to a given prediction by treating features as players in a coalition game.

The model’s output is represented by the Shapley value, which fairly attributes the prediction to each feature while maintaining consistency between local and global interpretations [27,28].

In this article, we focus on exploring the chemical-sensory profiles of single-origin coffee samples from five provenances through machine learning approaches. Specifically, we apply a Support Vector Machine (SVM) model to classify samples based on their volatile compound composition obtained via GC–MS analysis. Model interpretability is investigated using SHAP analysis, with the aim of identifying the key molecular features that characterise each coffee origin and define its specific aroma blueprint. Furthermore, a multivariate regression was applied to link GC–MS data with the coffee aroma intensity scale, aiming to provide a fast and objective tool for product quality evaluation and for assessing concordance with the industrial reference.

The central hypothesis of this work, which should be interpreted as a proof-of-concept, is that integrating predictive modelling with interpretability and sensory correlation can provide an objective and rapid framework for assessing coffee quality and for elucidating the molecular compounds that define its sensory identity. The objective is not to develop a fully validated analytical method for routine quantification of aroma intensity, but rather to investigate the relationships between chemical profile and sensory descriptors in an industrial context.

## 2. Materials and Methods

### 2.1. Coffee Samples

Samples of different origins of commercial roast and ground (R&G) coffees were supplied by Illycaffè S.p.A. (Trieste, Italy), including 32 coffees for moka preparation, packed in inert atmosphere under pressure in 125 g stainless steel cans (Table 1). Samples were stored at −18 °C until analysis. The study included five Brazilian, six Colombian, seven Ethiopian, seven Guatemalan, and seven Indian coffee samples from different industrial lots with various expiration dates. The aroma intensity values used in this study correspond to the intensity scale reported on product packaging, which reflects the internal sensory classification adopted by the company. These values are derived from routine evaluations performed by an internal industrial sensory panel for product positioning and consumer communication purposes. The scale ranges from 1 (mild intensity) to 9 (very strong intensity). It should be noted that this scale represents the aggregated outcome of the company’s internal sensory classification system. In the present study, it was therefore used as an ordinal descriptor of relative aroma intensity across samples, rather than as a quantitative sensory measurement.

### 2.2. Reference Standards and Solvents

Reference standards of n-alkanes (*n*-C9–*n*-C25) from Sigma-Aldrich (Milan, Italy) were used to determine the linear retention index (*I*^T^_S_). A standard stock solution of ISTDs (*n*-C13, 1000 mg/L) was prepared in dibutyl phthalate (Sigma-Aldrich, Milan, Italy) and stored in a sealed vial at −18 °C.

### 2.3. Sampling Conditions

Automated HS-SPME was performed using a MPS multipurpose sampler (Gerstel, Mülheim a/d Ruhr, Germany) installed on the GC-MS system. Five hundred mg of coffee R&G roasted powder in 20 mL vial were sampled by SPME fibres, Divinylbenzene/carboxen/polydimethylsiloxane (DVB/CAR/PDMS) (df 50/30 μm, 2 cm), were from Supelco (Bellefonte, PA, USA). Samples were incubated at 50 °C under agitation at 250 rpm to promote equilibration between the sample matrix and the headspace. The extraction was carried out for 40 min using the SPME fibre exposed to the headspace. Subsequently, thermal desorption of the analytes was performed in the GC injector for 300 s. Fibres were conditioned before being used as suggested by the manufacturer. 5 μL of ISTD (n-C13) at 1000 mg/L was placed in a 20 mL vial for headspace analysis. Two ISTDs were used in each tray of samples (32 samples) for normalising the analytical chromatographic responses.

Extracted analytes were recovered by thermal desorption of the fibre into the split/splitless (S/SL) in the injection port at 250 °C for 5 min in splitless mode. Each sample was analysed in duplicate. Injections for linear retention index (*I*^T^_S_) determination were carried out by manual injection: injection mode split; split ratio, 1:20; volume, 1 µL; and temperature, 250 °C.

### 2.4. GC-MS Instrument Setup

The GC-MS system consisted of an Agilent 7890A-GC system and a 5975C inert MSD operating in EI mode at 70 eV. The GC transfer line was set at 250 °C, and the ion source temperature at 230 °C. The scan range was set to *m*/*z* 35–350 with a scan speed of 666 amu/s. A SolGel-WAX column (100% polyethylene glycol) (30 m × 0.25 mm dc, 0.25 µm df) from Trajan (Trajan, Melbourne, Australia) was used. The carrier gas was helium, which was used at a constant flow rate of 1 mL/min. The oven temperature programme was 40 °C (1 min) to 200 °C at 3 °C/min, then to 250 °C (5 min) at 10 °C/min.

### 2.5. Data Acquisition and Statistical Analysis Software

Data from the GC-MS system were acquired with an MSD ChemStation E.02.01.1177. (Agilent Technologies, Inc., Santa Clara, CA, USA).

Multivariate data analysis was performed using Python code (version 3.11.8) within a Jupyter Notebook environment (v. 1.117). The following packages were used in the modules: NumPy (v. 2.4.0) [29], Pandas (v. 2.3.3) [30], Matplotlib (v. 3.10.1) [31], Plotly (v. 6.6.0) [32], Scikit-Learn (v. 1.8.0) [33] and SHAP (v. 0.51.0) [34]. Moreover, regression models were performed using MATLAB (v. 2025b) by means of the Regression Toolbox for MATLAB [35].

## 3. Analytes Identification Criteria and Data Analysis

### 3.1. Identification Workflow and Statistical Modelling 

Volatile and semi-volatile analytes detected and sampled from the coffee headspace were either identified by comparing their mass spectra with those of commercial libraries (Wiley 7N, Nist 23 Mass Spectral Data) and in-house databases or reported in the literature. *I*^T^_S_ were taken as a complementary parameter to support identification, and experimental values were compared to tabulated values in NIST Chemistry WebBook (tolerance ± 20 units) (https://webbook.nist.gov/chemistry/ (accessed on 15 December 2025). Table 2 presents the identified compounds along with their experimental retention times, quantifier *m*/*z* values, and the *m*/*z* values of the two primary qualifier ions.

Prior to data analysis, chromatographic responses were normalised to the internal standard, log_10_ transformed, and autoscaled. Explorative data analysis by PCA was used to control the presence of outliers. To extract meaningful insights from the chromatographic data, SVM model and SHAP method were applied. Furthermore, a multivariate regression was performed to correlate the chromatographic profiles with the aroma intensity reported on packaging. It typically refers to a scale or descriptor that communicates the perceived strength or boldness of the coffee’s flavour, aroma, and sometimes its body or aftertaste. It is not a standardised measure, but rather a sensory guide for consumers [36]. Given the relatively limited dataset (n = 32), model validation was performed using cross-validation to maximise the use of available samples while reducing overfitting risk. Model performance was evaluated using cross-validation metrics (RMSECV and R^2^CV). Although the dataset size is relatively limited (32 samples), it reflects a realistic industrial scenario in which well-characterised single-origin coffees are analysed. The objective of the modelling approach was not to develop a fully generalisable predictive model, but rather to explore the relationship between volatile profiles and coffee origin/intensity and to identify chemically meaningful markers through explainable AI.

SVM works by finding the optimal decision boundary (a hyperplane) that best separates different classes in the data. It aims to maximise the margin, which is the distance between the hyperplane and the nearest data points from each class, known as support vectors. A larger margin generally leads to better generalisation and improved classification performance.

SVMs can also handle complex, non-linearly separable data using kernel functions. These functions transform the original data into a higher-dimensional space where a clear separation becomes possible. Common kernel functions include polynomial, radial basis function (RBF), and sigmoid kernels [37].

Ridge Regression (RR) is a statistical method used to improve the reliability of multiple linear regression models when predictor variables are strongly correlated, a situation known as multicollinearity. In ordinary regression, such correlation can cause the estimated coefficients to become unstable and excessively large, reducing the model’s accuracy. Ridge regression solves this by adding a non-negative penalty parameter to the estimation process. This penalty reduces the size of the coefficients, pulling them closer to zero, which lowers the variability of the estimates and improves overall accuracy, even though it introduces a small bias. The strength of the penalty is controlled by a tuning parameter, and analysing how coefficients change as this parameter varies, through a tool called the ridge trace, helps select the most suitable value. Ridge regression has a solid theoretical basis and can be understood in several ways, including as a constrained optimisation method or as an approach that balances different regression solutions. Its ability to produce stable and interpretable estimates makes it a powerful alternative to ordinary regression, particularly when dealing with highly correlated predictors [38].

### 3.2. SHAP Analysis: Theoretical Framework

XAI methods have been developed to enhance the interpretability of ML and DL models by clarifying how individual features contribute to model predictions. These approaches aim to transform complex “black-box” models into more transparent and trustworthy systems, providing both local and global explanations of their outputs. Among the various XAI techniques proposed, SHAP has emerged as one of the most widely adopted methods across different domains, offering a theoretically grounded framework for quantifying each feature’s contribution to a model’s predictions [39].

SHAP analysis is rooted in Shapley values, a concept from collaborative game theory. Shapley value aims to fairly distribute contributions of players when they collectively achieve a certain outcome. Shapley values can be used in machine learning to quantify the contribution of each feature in the model that collectively delivers the prediction [40].

Shapley [41] defined four key properties for a fair measure of contribution in a cooperative game:

- Efficiency: the sum of all players’ contributions must equal the total payout;
- Symmetry: players who contribute equally to all coalitions should receive equal payouts;
- Additivity: a player’s contribution to a combined game equals the sum of their contributions to each individual subgame;
- Null player: a player who contributes nothing to any coalition receives a payout of zero.

Shapley values can be calculated by considering a payout, *V*, each player *j* (chemical variable or feature) is assigned a Shapley value, *Φ_j_*, that corresponds to their fair share of the payout based on their individual contribution. The mathematical formula for *Φ_j_* is the following:

(1) $$\Phi_{j}=\sum_{S\subseteq N\backslash \{j\}}\frac{\left|S\right|!\left(\left|N\right|-\left|S\right|-1\right)!}{\left|N\right|-1}(V(S\cup \left\{j\right\})-V\left(S\right))\;$$

where *S* is a coalition (a subset of features), *N* is total number of features, $\left(V(S\cup \left\{j\right\})-V\left(S\right)\right)$ quantifies the marginal contribution of feature *j* to coalition *S*, $\frac{\left|S\right|!\left(\left|N\right|-\left|S\right|-1\right)!}{\left|N\right|-1}$ is the weight of the marginal contribution, indeed, $\left(V(S\cup \left\{j\right\}\right)$ is the model prediction with features in *S* plus feature *j* and ∑_*S*⊆*N*\{*j*}_ sums over all possible coalitions without *j*. The weights are the inverse of the number of coalitions of size |*S*| excluding player *j*. Shapley values can be interpreted as the weighted average of a player’s marginal contributions across all possible coalitions [40,42].

## 4. Results and Discussion

### 4.1. Modelling the Coffee Origin Identity

The preprocessed data, transformed by log_10_ and autoscaling, were used to explore the data and to train the SVM model. Exploratory data analysis does not reveal outliers in the samples analysed. Hyperparameter optimisation for SVM modelling was performed using the GridSearchCV function from the scikit-learn library, which systematically explores a predefined set of parameter values and evaluates model performance for each combination in order to identify the best configuration [43]. Specifically, the parameter grid included variations in kernel types (linear, RBF, polynomial), regularisation strengths (C = [0.1, 1, 10, 100]), and kernel-specific parameters such as gamma ([‘scale’, ‘auto’, 0.01, 0.001]), degree ([2–4]), and coef0 ([0.0, 0.1, 0.5]), along with different class weighting schemes ([None, ‘balanced’]).

The SVM model was initialised with default settings aside from the specified parameter grid. GridSearchCV was configured with three-fold cross-validation and accuracy as the scoring metric. This approach allowed for a systematic evaluation of each parameter combination to determine the configuration that maximised predictive performance. The optimal hyperparameters found were C = 1, kernel = ‘linear’, and class_weight = None, resulting in a mean cross-validation accuracy of 0.91 (Table 3).

Even though SVM is a powerful classification method, its nature as a ‘black box’ reduces its interpretability. As a result, the model itself does not provide any explanation of which features lead to a particular classification output [44]. For this reason, the SHAP method, based on the Permutation Explainer, was applied to interpret the classification results of the SVM model. The explainer was initialised using the model’s probability predictions, and SHAP values were computed for the input dataset. To focus on a specific classification outcome, only the values corresponding to the target class were extracted.

### 4.2. Defining the Chemical Boundaries of Coffee Origin Identity

**Figure 1:**
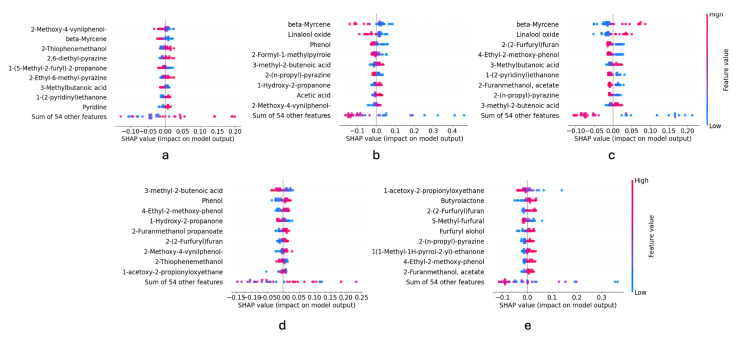
Beeswarm plot of (**a**) Brazil, (**b**) Colombia, (**c**) Ethiopia, (**d**) Guatemala, (**e**) India.

While SHAP does not provide mechanistic causality, it enables the identification of volatile compounds that systematically influence model predictions. In this context, SHAP serves as a bridge between chemometric modelling and chemical interpretation by highlighting the compounds that contribute most strongly to origin discrimination or intensity prediction. The SHAP beeswarm plot provides a global overview of SHAP values for selected features. Each row represents a feature, ranked by its mean absolute SHAP value. The individual dots in a row correspond to the SHAP values of that feature for each sample in the dataset. Additionally, the plot reveals whether low or high feature values contribute positively to the classification (a positive impact means the model is more likely to classify the sample into the target class) [42]. Figure 1 shows the beeswarm plot of each coffee origin.

Based on this, for the Brazil class, high values of 2-thiophenemethanol are associated with a positive impact on the prediction of the Brazil class, meaning that this compound makes the model more likely to classify a sample as Brazilian. Similarly, high values of pyridine, 2,6-diethyl-pyrazine, 2-ethyl-6-methyl-pyrazine and 1-(2-pyridynil)ethanone, push the prediction towards this class. In contrast, a high value of 2-methoxy-4-vinylphenol tends to reduce the probability of Brazil.

β-myrcene and linalool oxide exhibit opposite effects in the Colombia and Ethiopia classes: high values of these compounds increase the probability of classification as Ethiopia, while low values favour classification as Colombia. Similarly, high values of 2-furanmethanol propanoate favour the classification for the Guatemala class, while low values for Ethiopia. Moreover, high values of 1-acetoxy-2-propionyloxyethane favour the classification for Guatemala, while low values for India.

Many of the compounds identified as influential by SHAP belong to chemical families known to arise during Maillard reactions and thermal degradation processes occurring during roasting, such as pyrazines, furans, and pyrroles. These compounds are widely recognised as key contributors to roasted, nutty, and caramel-like sensory attributes in coffee. Their differential abundance across origins may reflect variations in precursor composition, including amino acids and sugars, which are influenced by cultivar, terroir, and post-harvest processing.

**Figure 2:**
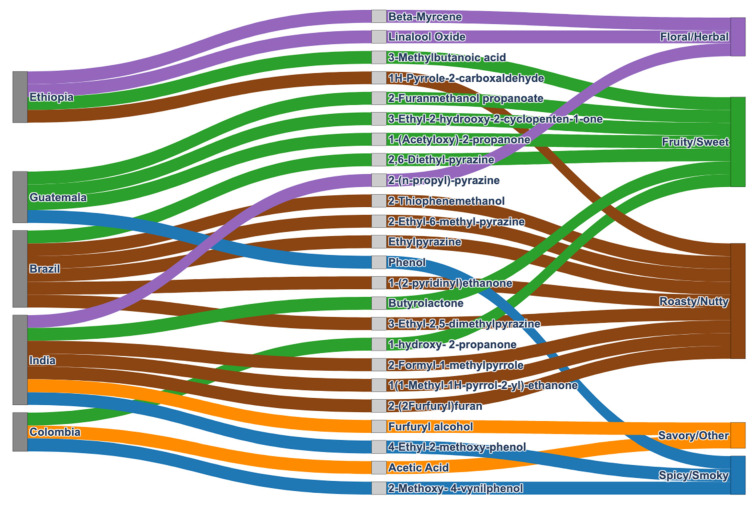
Sankey plot of key volatile compounds identified as contributors to origin discrimination by SHAP analysis and their associated flavour descriptors from the literature [45].

Based on the SHAP analysis, the compounds identified as key contributors to the discrimination of a specific origin were correlated with their associated flavours (Figure 2, Table S1).

Brazilian samples were dominated by pyrazines, such as 2-ethyl-6-methyl pyrazine, and 3-ethyl-2,5-dimethyl pyrazine, which are well-known to contribute to roasted, nutty, and cocoa-like notes together with some sweet flavours. Due to their very low odour thresholds, they strongly shape overall aroma even at low concentrations [46]. Colombian samples exhibited the presence of acetic acid and 2-methoxy-4-vinylphenol, which may be linked to acidic and smoky-clove attributes, while 1-hydroxy-2-propanone could have added mild sweet-green undertones [47]. Ethiopian samples were distinguished by terpenes, in particular linalool oxide, which may have imparted floral, herbal, and balsamic notes, along with β-myrcene, associated with geranium-like and hop-like nuances. In particular, monoterpenes dominate the volatile profile in light roasting, but as temperature increases, Maillard-derived compounds rapidly become dominant in the aroma profile [48]. This combination of compounds could suggest a delicate aromatic profile. Indian samples were characterised by high levels of 4-ethyl-2-methoxy phenol, responsible for spicy and smoky attributes, complemented by pyrazines and pyrroles that could impact on roasted, nutty, and earthy notes. In Guatemalan samples, cyclopentenones and furan derivatives contribute to the overall sensory profile. Furans are among the most abundant coffee volatiles and are strongly associated with roasted and caramel-like aroma notes, while cyclopentenones, though less prominent, belong to a broader group of lactones and related compounds that further modulate aroma perception [49].

While this evaluation highlights the main compounds contributing to each sample’s aroma traits, it is important to note that the overall sensory profile cannot be explained solely by the effects of individual molecules. In this study, the contribution of each volatile compound was considered individually; however, aroma complexity in foods and beverages primarily arises from the interactions among numerous volatile organic compounds (VOCs), including esters, terpenes, aldehydes, alcohols, lactones, and sulphur-containing compounds. Although hundreds of VOCs may be present, only a subset significantly influences perceived aroma. The final profile is determined not by any single compound, but by the combination and relative proportions of the various molecules. Moreover, aroma perception is not simply additive: interactions between VOCs can generate new sensory qualities or modulate the intensity of existing notes. Certain compounds can affect the perception of others, introducing unexpected aromatic nuances. Synergistic or antagonistic interactions among molecules further complicate the sensory outcome, making it difficult to directly associate specific compounds with particular aroma notes [50,51].

### 4.3. Modelling the Aroma Intensity

Regarding the modelling of aroma intensity reported on the packaging, the RR was employed. As aforementioned, this approach was selected for its ability to handle the multicollinearity typical of chromatographic data and to provide robust coefficient estimation. In this case, the chromatographic responses were used as independent variables, while aroma intensity was treated as the dependent variable, as defined by the company on a scale from 1 to 9. The intensity values for the different origins are: Brazil 5, Colombia 4, Ethiopia 3, Guatemala 6, and India 8.

**Figure 3:**
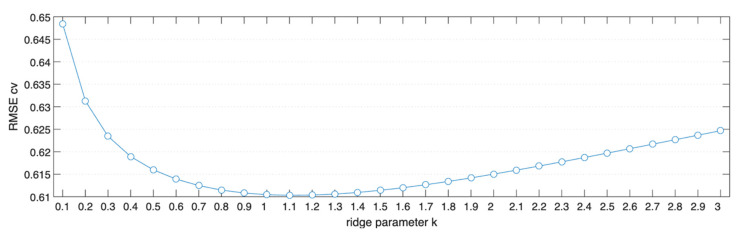
Optimal k value for the Ridge Regressor.

The optimal k value was determined by means of cross-validation, taking into account the Root Mean Square Error RMSE. Based on this, a k value equal to 1 was chosen for the regressor (Figure 3).

Then, the model has been trained and tested by a three-fold cross-validation, reaching the following performances (Table 4):

The Ridge Regressor was trained using three-fold cross-validation. The model achieved strong predictive performance, with RMSE = 0.26 on the training set and RMSE = 0.61 on cross-validation, and R^2^ = 0.98 and 0.88, respectively.

On the 1–9 intensity scale, this corresponds to an average deviation of about half a point, which may lead to partial overlap between adjacent intensity levels (e.g., 3 and 4).

**Figure 4:**
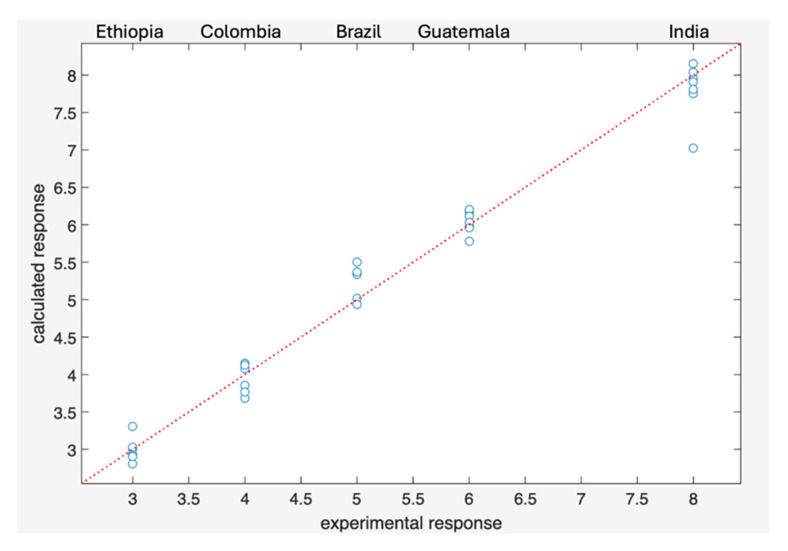
Experimental response vs. calculated response based on cross-validation.

One Indian sample was identified as an outlier, showing a lower predicted intensity compared to the rest of the group (Figure 4). After its removal, the cross-validated RMSE decreased to 0.38, substantially improving predictive accuracy and eliminating the overlap between intensity levels.

**Figure 5:**
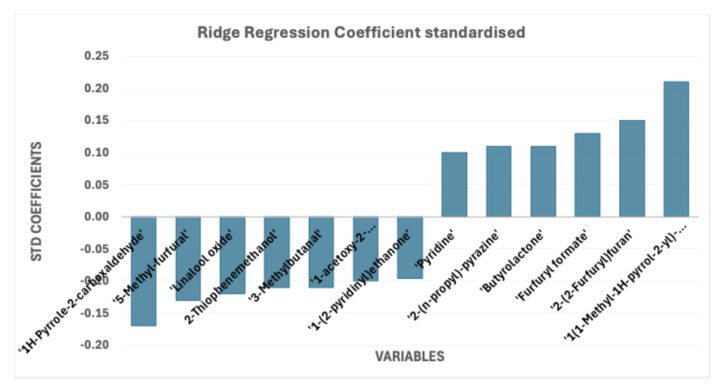
Ridge Regression coefficient standardised.

From the RR analysis, the coefficients were extracted to further investigate the molecules influencing aroma intensity (Figure 5). Compounds with high RR coefficients are particularly important for achieving a high level of aroma intensity, as they contribute significantly to its increase. Indeed, an increase in their concentration corresponds to a higher perceived aroma intensity. The key compounds identified are pyridine, 2-(n-propyl)-pyrazine, butyrolactone, furfuryl formate, 2-(2-furfuryl)furan, and 1-(1-methyl-1H-pyrrol-2-yl)ethenone.

On the other hand, an increase in the concentration of 1-(2-pyridinyl)ethanone, 1-acetoxy-2-propionyloxyethane, 3-methylbutanal, 2-thiophenemethanol, linalool oxide, 5-methylfurfural, and 1H-pyrrole-2-carboxaldehyde was associated with a decrease in aroma intensity.

Our results are in line with those already provided by different authors in previous works. Ruosi et al. [6] observed that pyridine concentration increases with higher roasting levels, whereas 5-methylfurfural decreases as roasting progresses, and 3-methylbutanal remains relatively stable across different roasting levels. Linalool and its oxides are most abundant in light and medium roasts [6,52]. Butyrolactone increases with roasting intensity, peaking in medium to dark roasts, while 1-methyl-1H-pyrrole-2-carboxaldehyde increases up to medium roast levels and then decreases in dark roasts [53]. The compound concentration of 2-(2-furfuryl)furan increases with roasting temperature [54]. Pyrrole derivatives, such as 1-(1-methyl-1H-pyrrol-2-yl)ethanone, also increase with roasting, being more prevalent in medium and dark roasts and nearly absent in light roasts [55]. Finally, 1-acetoxy-2-propionyloxyethane is more abundant in light to medium roasts but tends to decrease with darker roasting [15].

In summary, the combined application of SVM classification and Ridge Regression, complemented by SHAP-based interpretability, enabled the reliable discrimination of coffee origins and accurate prediction of aroma intensity. While SVM effectively captured the origin-specific chemical signatures and highlighted key volatile compounds driving classification, Ridge Regression based on coffee profile robustly modelled intensity despite multicollinearity in chromatographic data. Together, these approaches provide both predictive power and mechanistic insights, demonstrating how chemometric and explainable artificial intelligence can deepen understanding of coffee chemical and sensory profiles.

## 5. Conclusions

Specialty coffee is valued for its unique quality, distinctive sensory profile, and traceable origin. Terroir, reflecting the interaction between cultivation practices and local environmental conditions, plays a crucial role in shaping both consumer perception and product differentiation. Along with terroir, coffee aroma intensity emerges as a key sensory attribute, influencing consumer preference and expectations, and is closely linked to both chemical composition and roasting practices.

In this study, we demonstrated that volatile profiles obtained via HS-SPME/GC–MS, when analysed through machine learning, can accurately capture both coffee origin and sensory intensity. The SVM classification model achieved high accuracy (0.91) in discriminating coffee origins, providing a reliable tool for industrial quality control. Explainable AI (SHAP) further allowed the identification of characteristic compounds for each origin, enabling the tracing of origin-specific sensory profiles directly from machine learning outputs. Finally, the multivariate regression model was used to link the chemical profile to aroma intensity. This approach enhances the transparency and credibility of the information conveyed to consumers, reinforcing the perception that the declared aroma intensity reflects objective product characteristics rather than purely subjective assessments. It also provides an additional tool for the objective monitoring of sensory quality. This study presents an integrated framework combining predictive modelling, interpretability, and chemical–sensory correlations for quality control and marketing specialty coffee. The approach enables objective monitoring of coffee quality, ensures consistency in sensory classification, and aligns with expert panel evaluations, supporting traceability, reproducibility, and high sensory standards.

Although the dataset size is relatively limited (32 samples), it reflects a realistic industrial scenario in which well-characterised single-origin coffees are analysed. The objective was to evaluate the feasibility of combining volatile profile with explainable machine learning rather than to build a universally generalizable classifier. Future studies involving larger datasets and additional origins will allow further validation and extension of the proposed approach. Indeed, the framework demonstrates how analytical chemistry, machine learning, and explainable AI can be effectively integrated to obtain robust and interpretable results, offering a practical strategy for supporting data-driven quality control and decision-making in industrial coffee production.

## Figures and Tables

**Table 1:** Sample ID and sensory intensity.

Sample ID	Lot Number	Expiring Data	Origin	Intensity Score
Brazile_1	231122	Nov-24	Brazil	5
Brazile_2	081123	Nov-25	Brazil	5
Brazile_3	250624	Jun-26	Brazil	5
Brazile_4	061224	Dec-26	Brazil	5
Brazile_5	270924	Sep-26	Brazil	5
Colombia_1	190224	Feb-26	Colombia	4
Colombia_2	180123	Jan-25	Colombia	4
Colombia_3	150323	Mar-25	Colombia	4
Colombia_4	271123	Nov-25	Colombia	4
Colombia_5	170125	Jan-27	Colombia	4
Colombia_6	290823	Aug-25	Colombia	4
Ethiopia_1	150323	Mar-25	Ethiopia	3
Ethiopia_2	130923	Sep-25	Ethiopia	3
Ethiopia_3	70225	Feb-27	Ethiopia	3
Ethiopia_4	241123	Nov-25	Ethiopia	3
Ethiopia_5	240723	Jul-25	Ethiopia	3
Ethiopia_6	250124	Jan-26	Ethiopia	3
Ethiopia_7	281124	Nov-26	Ethiopia	3
Guatemala_1	200323	Mar-25	Guatemala	6
Guatemala_2	030124	Jan-26	Guatemala	6
Guatemala_3	201123	Nov-25	Guatemala	6
Guatemala_4	150524	May-26	Guatemala	6
Guatemala_5	220823	Aug-25	Guatemala	6
Guatemala_6	161224	Dec-26	Guatemala	6
Guatemala_7	100225	Feb-27	Guatemala	6
India_1	210323	Mar-25	India	8
India_2	030823	Aug-25	India	8
India_3	260723	Jul-25	India	8
India_4	310724	Jul-26	India	8
India_5	70223	Feb-25	India	8
India_6	111124	Nov-26	India	8
India_7	230224	Feb-26	India	8

**Table 2:** List of analytes with their retention times, experimental and literature Retention Index (*I*^T^_S_), similarity search index (SI), Target ion (Ti) *m*/*z* values, and the *m*/*z* values of the two primary qualifier ions (Q and Q2). * *I*^T^_S_ extrapolated.

Compound	CAS No.	Rt	Expl *I*^T^_S_	Lit *I*^T^_S_	SI	*m*/*z* Ti	*m*/*z*-Q1	*m*/*z*-Q2
Acetone	67-64-1	2.21	835 *	823	95	58	43	59
Methyl acetate	79-20-9	2.26	840 *	828	85	43	59	74
2-Methylfuran	534-22-5	2.55	868 *	868	94	82	51	53
2-Butanone	78-93-3	2.82	891 *	899	90	43	57	72
2-Methylbutanal	96-17-3	2.94	900 *	907	96	57	41	55
3-Methylbutanal	590-86-3	2.99	904 *	909	98	44	58	71
2,5-Dimethylfuran	625-86-5	3.43	936 *	939	95	96	53	95
2,3-Pentanedione	600-14-6	5.38	1039 *	1035	98	43	57	100
2-Vinylfuran	1487-18-9	5.67	1051 *	1051	94	43	41	71
2,3-Hexanedione	3848-24-6	7.27	1108	1136	94	43	41	71
1-Methyl-1H-pyrrole	96-54-8	7.45	1114	1139	96	81	53	78
3,4-Hexanedione	4437-51-8	7.53	1116	1123	88	57	81	114
2-Vinyl-5-methylfuran	10504-13-9	7.91	1128	1127	89	108	65	79
β-Myrcene	123-35-3	8.25	1137	1140	97	93	41	69
Pyridine	110-86-1	8.71	1151	1160	99	79	51	52
Methylpyrazine	109-08-0	11.73	1226	1235	94	94	43	67
3-Hydroxy-2-butanone	513-86-0	12.43	1244	1250	94	45	43	88
2,7-dimethyloxepine	1487-99-6	12.72	1252	-	90	122	77	43
1-Hydroxy-2-propanone	116-09-6	13.05	1260	1275	93	43	45	74
2,5-Dimethylpyrazine	123-32-0	13.87	1280	1290	98	42	43	108
2,6-Dimethylpyrazine	108-50-9	14.13	1286	1298	97	108	40	42
Ethylpyrazine	13925-00-3	14.32	1290	1302	96	108	80	107
2,3-dimethylpyrazine	5910-89-4	14.79	1301	1306	98	108	40	67
2-Hydroxy-3-pentanone	5704-20-1	15.32	1316	1353	82	45	57	71
2-Methyl-2-cyclopenten-1-one	1120-73-6	15.56	1323	1355	94	96	53	67
2-Ethyl-6-methylpyrazine	13925-03-6	16.39	1344	1353	98	122	94	121
2-Ethyl-5-methylpyrazine	13360-64-0	16.60	1349	1358	97	121	39	122
2-Ethyl-3-methylpyrazine	15707-23-0	17.18	1363	1363	91	42	121	122
2-(n-propyl)-pyrazine	18138-03-9	17.67	1375	1404	91	94	107	122
2,6-Diethylpyrazine	13067-27-1	18.41	1392	1415	76	135	71	136
Acetic acid	64-19-7	18.69	1398	1403	97	43	45	60
Linalool oxide	5989-33-3	18.77	1401	1420	94	59	94	111
5-Ethyl-2,5-dimethylpyrazine	15707-34-3	18.87	1408	1420	96	135	56	121
3-Ethyl-2,3-dimethylpyrazine	13360-65-1	19.45	1420	1430	95	135	136	108
Furfural	98-01-1	19.71	1427	1432	98	96	67	95
1-(Acetyloxy)-2-propanone	592-20-1	19.99	1435	1454	97	43	86	116
2-ethenyl-5-methylpyrazine	13925-08-1	20.50	1449	1468	89	120	52	119
3,5-diethyl-2-methylpyrazine	18138-05-1	20.75	1456	1469	82	149	120	150
Furfuryl formate	13493-97-5	21.09	1465	1478	92	126	53	81
1-(2-furanyl)-ethanone	1192-62-7	21.19	1467	1479	97	95	96	110
Benzaldehyde	100-52-7	21.72	1481	1488	90	106	51	77
2,3-Dimethyl-2-cyclopenten-1-one	1121-05-7	22.26	1494	1524	90	67	95	110
1-Acetoxy-2-propionyloxyethane	98962-89-1	22.47	1499	1486	90	57	87	100
2-Furanmethanol acetate	623-17-6	22.74	1507	1514	98	98	140	57
5-Methylfurfural	620-02-0	23.98	1538	1536	97	110	109	53
1-(2-Pyridinyl)ethanone	1122-62-9	24.81	1561	1560	90	79	121	78
2-Furanmethanol propanoate	623-19-8	25.03	1568	1587	71	57	81	98
2-(2-Furfuryl)furan	1197-40-6	25.37	1580	1588	94	148	91	119
2-Formyl-1-methylpyrrole	1192-58-1	25.50	1587	1606	96	109	53	108
Butyrolactone	96-48-0	26.68	1616	1617	89	56	41	42
1(1-methyl-1H-pyrrol-2-yl)ethanone	932-16-1	26.82	1635	1645	93	108	53	123
Furfuryl alcohol	98-00-0	27.44	1640	1651	97	98	69	81
3-Methyl-butanoic acid	503-74-2	27.66	1656	1666	95	60	41	43
2(5H)-Furanone	497-23-4	30.30	1710	1716	81	55	54	84
1-(1,2-dimethylcyclopent-2-en-1-yl)ethanone	70987-82-5	31.38	1741	-	87	95	53	138
3-Methyl-2-butenoic acid,	541-47-9	32.22	1766	1776	87	100	55	82
3-Ethyl-2-hydroxy-2-cyclopenten-1-one	21835-01-8	35.43	1859	1891	94	126	55	83
2-Thiophenemethanol	636-72-6	37.10	1908	1917	80	114	85	97
1-(1H-pyrrol-2-yl)-ethanone	1072-83-9	37.96	1935	1949	96	94	66	109
Phenol	108-95-2	39.20	1972	1973	81	94	39	66
1H-Pyrrole-2-carboxaldehyde	1003-29-8	39.59	1984	1978	95	95	39	94
4-Ethyl-2-methoxy-phenol	2785-89-9	39.97	1994	1987	86	137	94	152
2-Methoxy-4-vinylphenol	7786-61-0	45.08	2161	2156	97	135	107	150

**Table 3:** Confusion matrix based on cross-validation with 3 folds.

	Precision	Recall	F1-Score
Brazil	0.71	1.00	0.83
Colombia	1.00	0.83	0.91
Ethiopia	1.00	1.00	1.00
Guatemala	0.86	0.86	0.86
India	1.00	0.86	0.92
	Model Accuracy		0.91

**Table 4:** Performances of Ridge Regressor.

Measure	Training	CV
RMSE	0.26	0.61
R^2^	0.98	0.88

## Data Availability

The raw data supporting the conclusions of this article will be made available by the authors on request.

## Supplementary Materials

The following supporting information can be downloaded at: https://www.mdpi.com/article/10.3390/foods15091543/s1, Table S1. List of key volatile compounds identified as contributors to origin discrimination by SHAP analysis and their associated flavour descriptors from the literature.
