# Supplementary material for: Explainable Artificial Intelligence for Coffee Quality Control: From Coffee Origins to Aroma Intensity
Source: Foods. 2026 Apr 29;15(9):1543. doi: 10.3390/foods15091543 (PMC13163920; doi:10.3390/foods15091543)
Supplement: Supplementary file 1 [file foods-15-01543-s001.zip › foods-4235043-supplementary.pdf]

Explainable Artificial Intelligence for Coffee Quality Control: from Coffee Origins to Aroma Intensity

Giorgio Felizzato[1], Eloisa Bagnulo[1], Giorgia Botta[1], Giulia Tapparo[1], Chiara Cordero[1], Luciano Navarini[2], Cecilia Cagliero[1], Erica Liberto[1*], Andrea Caratti[1]

[1] Università di Torino Dipartimento di Scienza e Tecnologia del Farmaco, Via Pietro Giuria 9, Italy
[2] Illycaffè S.p.A., via Flavia 110, Trieste I-34147, Italy

Table S1 List of key volatile compounds identified as contributors to origin discrimination by SHAP analysis and their associated flavour descriptors from the literature (Kreissl J, 2022).

Compound	Flavour
Brazil	
2-Thiophenemethanol	Savory, coffee, roasty
Pyrazine, 2-ethyl-6-methyl-	Roasty, nutty
Pyrazine, 2,6-diethyl-	Sweet
Pyrazine, ethyl-	Roasty, rum-like
Ethanone, 1-(2-pyridinyl)	Roasty
Pyrazine, 3-ethyl-2,5-dimethyl	Nutty, cocoa
Colombia	
Acetic Acid	Vinegar-like
2-Methoxy-4-vynilphenol-	Spicy, smoky, clove-like
2-Propanone, 1-hydroxy-	Sweet, green
Ethiopia	
Beta-Myrcene	Geranium-like, carrot-like, hop-like
Linalool Oxide	Floral, herbal, balsamic
Butanoic acid, 3-methyl	Sweet
1H-Pyrrole-2-carboxaldehyde	Musty, coffee
Guatemala	
Phenol	ink-like, phenolic
2-Furanmethanol, propanoate	Fruity
2-Cyclopenten-1-one, 3-ethyl-2-hydrooxy-	Sweet, fruity, caramellic, jammy
2-Propanone, 1-(acetyloxy)-	Fruity, buttery
India	
Pyrazine, 2-(n-propyl)-	Green, vegetable
Butyrolactone	sweet, aromatic
2-Formyl-1-methylpyrrole	Roasted, nutty
Ethanone, 1(1-methyl-1H-pyrrol-2-yl)-	Nutty, floral. earthy
2-(2Furfuryl)furan	Roasted, aromatic
Phenol, 4-ethyl-2-methoxy-	Spicy, smoky
Furfuryl alcohol	Cooked ham-like, sweaty
